# Supplementary material for: Aphidius colemani Behavior Changes Depending on Volatile Organic Compounds Emitted by Plants Infected with Viruses with Different Modes of Transmission
Source: Insects. 2024 Jan 29;15(2):92. doi: 10.3390/insects15020092 (PMC10889700; doi:10.3390/insects15020092)

Aphidius colemani behavior Changes Depending on Volatile Organic Compounds Emitted by Plants Infected with Viruses with Different Modes of Transmission. Clemente-Orta, G., Cabello, A., Garzo, E., Moreno, A., Fereres, A.

Supplementary material

Table S1. Treatments used in olfactometry assays. Statistical results regarding olfactometry assays are shown.

n	Treatment		Melon cv.	Virus infection	Aphids infestation	Sex	Election	No election	chi2	*df*	*p value*
1	Control	Healthy C311 Vs. Healthy Bazán	C311 and Bazán	no	no	males	26	35	0.15	1	0.694
2						females	28	36	0	1	1
3		Air Vs. Healthy Bazán	Bazán	yes	no	males	26	34	0.15	1	0.694
4						females	49	66	0.02	1	0.886
5	CMV	Air Vs. CMV	Bazán	yes	no	males	25	32	0.04	1	0.815
6						females	29	37	5.82	1	**0.015**
7		Mock Vs. CMV	Bazán	yes	no	males	20	26	0.2	1	0.654
8						females	14	50	0.61	1	0.434
9	CABYV	Air Vs. CABYV	Bazán	yes	no	males	21	28	1.19	1	0.275
10						females	21	24	0.42	1	0.512
11		Mock Vs. CABYV	Bazán	yes	no	males	24	24	0.04	1	0.827
12						females	22	24	0.18	1	0.669
13	Mixed	Air Vs. mixed	Bazán	yes	no	males	23	26	0.39	1	0.531
14						females	29	33	12.44	1	**0.0004**
15		Mock Vs. mixed	Bazán	yes	no	males	26	32	0.15	1	0.694
16						females	23	32	0.39	1	0.531
17	Control	Air Vs. Healthy C311	C311	yes	no	males	23	28	1.08	1	0.297
18						females	20	25	3.2	1	0.07
19	CMV	Air Vs. CMV	C311	yes	no	males	21	25	5.76	1	**0.016**
20						females	21	25	0.81	1	0.369
21		Mock Vs. CMV	C311	yes	no	males	21	25	1.19	1	0.275
22						females	22	31	1.63	1	0.2
23	CABYV	Air Vs. CABYV	C311	yes	no	males	21	21	0.42	1	0.512
24						females	20	22	0.2	1	0.654
25		Mock Vs. CABYV	C311	yes	no	males	20	25	0	1	1
26						females	27	31	0.9	1	0.335
27	Mixed	Air Vs. mixed	C311	yes	no	males	23	33	0.04	1	0.834
28						females	22	30	0.18	1	0.669
29		Mock Vs. mixed	C311	yes	no	males	20	26	0.2	1	0.6547
30						females	22	26	0.18	1	0.669
31	Control	Healthy C311 Vs. Healthy Bazán	C311 and Bazán	no	yes	females	31	40	5.45	1	**0.02**
32		Air Vs. Healthy Bazán	Bazán	yes	yes	males	30	44	1.2	1	0.273
33						females	30	38	6.53	1	**0.01**
34		Healthy + aphids Vs. Healthy Bazán	Bazán	yes	yes	females	31	40	7.25	1	**0.007**
35	CMV	Air Vs. CMV	Bazán	yes	yes	females	31	40	0.81	1	0.369
36		Mock Vs. CMV	Bazán	yes	yes	females	30	40	0.53	1	0.465
37	CABYV	Air Vs. CABYV	Bazán	yes	yes	females	33	40	1.48	1	0.223
38		Mock Vs. CABYV	Bazán	yes	yes	females	48	60	8.02	1	**0.004**
39		CABYV + aphids Vs. CABYV	Bazán	yes	yes	females	32	40	0.12	1	0.723
40	Mixed	Air Vs. mixed	Bazán	yes	yes	females	31	40	0.03	1	0.857
41		Mock Vs. mixed	Bazán	yes	yes	females	30	40	0.13	1	0.715
42	Control	Air Vs. Healthy C311	C311	yes	yes	males	32	44	1.12	1	0.288
43						females	34	40	11.76	1	**0.0006**
44		Healthy + aphids Vs. Healthy C311	C311	yes	no	females	48	57	3	1	0.08
45	CMV	Air Vs. CMV	C311	yes	yes	females	36	40	5.44	1	**0.01**
46		Mock Vs. CMV	C311	yes	yes	females	39	49	3.1	1	0.007
47	CABYV	Air Vs. CABYV	C311	yes	yes	females	42	50	6.09	1	**0.013**
48		Mock Vs. CABYV	C311	yes	yes	females	40	50	0.9	1	0.342
49		CABYV c311 Vs. CABYV Bazán	C311 and Bazán	yes	yes	females	33	41	0.87	1	0.601
50		CABYV + aphids Vs. CABYV	C311	yes	yes	females	31	56	0.03	1	0.857
51	Mixed	Air Vs. mixed	C311	yes	yes	females	42	50	6.09	1	**0.013**
52		Mock Vs. mixed	C311	yes	yes	females	40	50	0.9	1	0.342

Aphidius colemani behavior Changes Depending on Volatile Organic Compounds Emitted by Plants Infected with Viruses with Different Modes of Transmission. Clemente-Orta, G., Cabello, A., Garzo, E., Moreno, A., Fereres, A.

Figure S1. Treatment photos used in olfactometry assays using both varieties of melon.

**Cv. Bazán, treatment: Mock (left) Vs. CMV (right)**
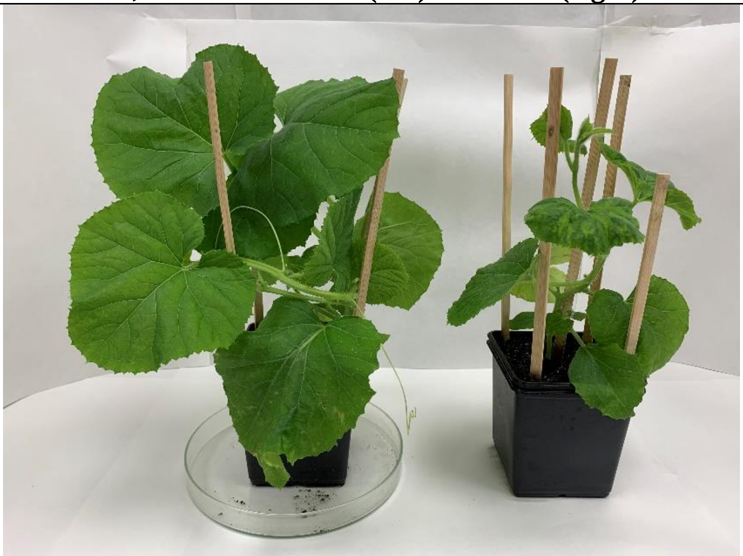
**Cv. Bazán, treatment: Mock (left) Vs. CABYV (right)**
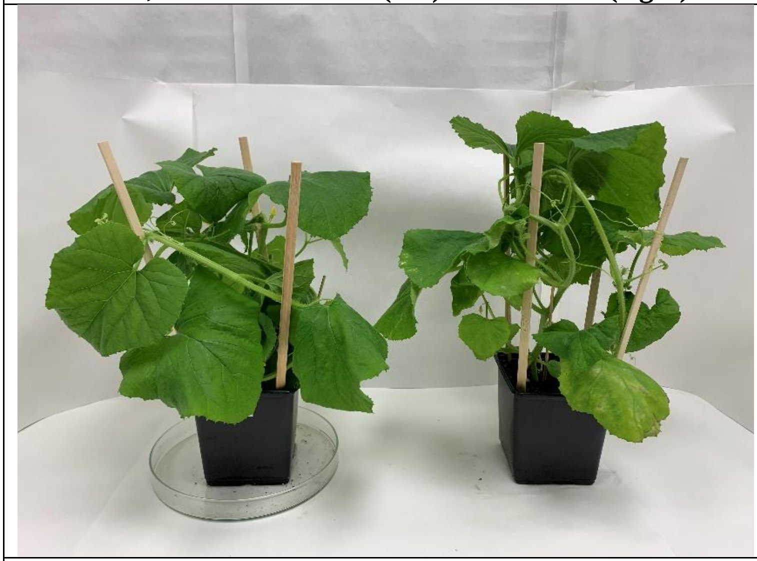
**Cv. Bazán, treatment: Mock (left) Vs. Mix (right)**
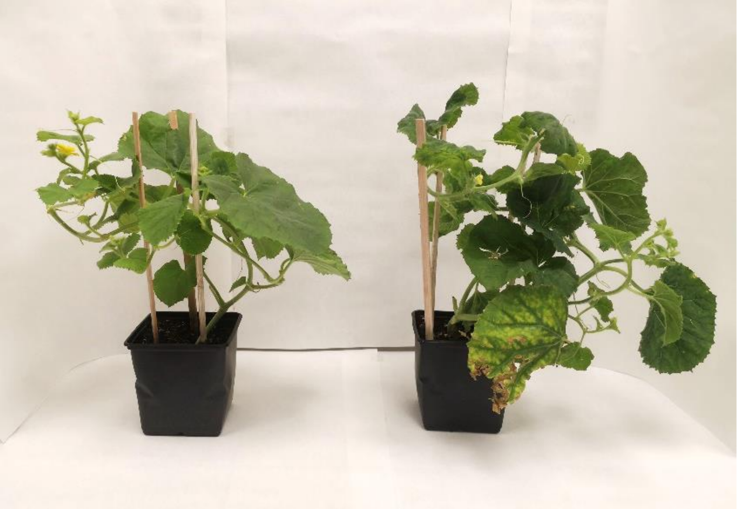
**C311, treatment: Mock (left) Vs. CMV (right)**

Aphidius colemani behavior Changes Depending on Volatile Organic Compounds Emitted by Plants Infected with Viruses with Different Modes of Transmission. Clemente-Orta, G., Cabello, A., Garzo, E., Moreno, A., Fereres, A.

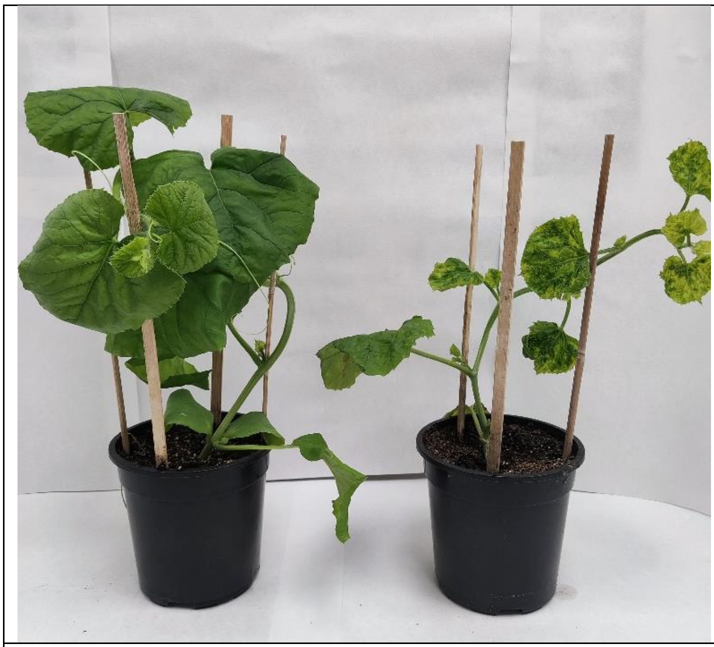

C311, treatment: Mock (left) Vs. CABYV (right)

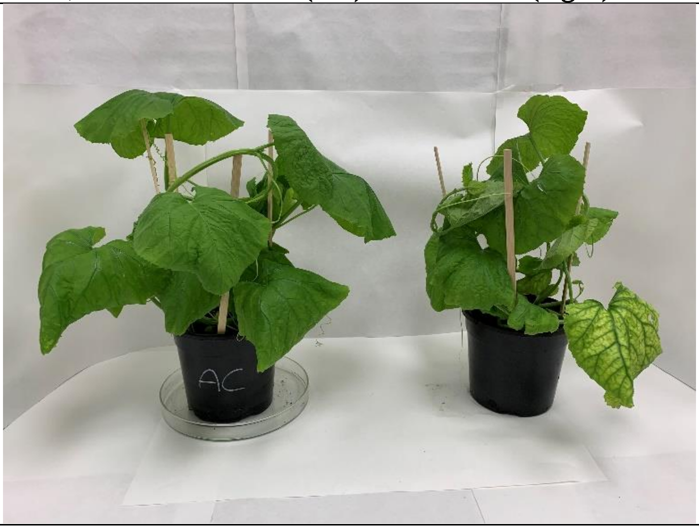

C311, treatment: Mock (left) Vs. Mix (right)

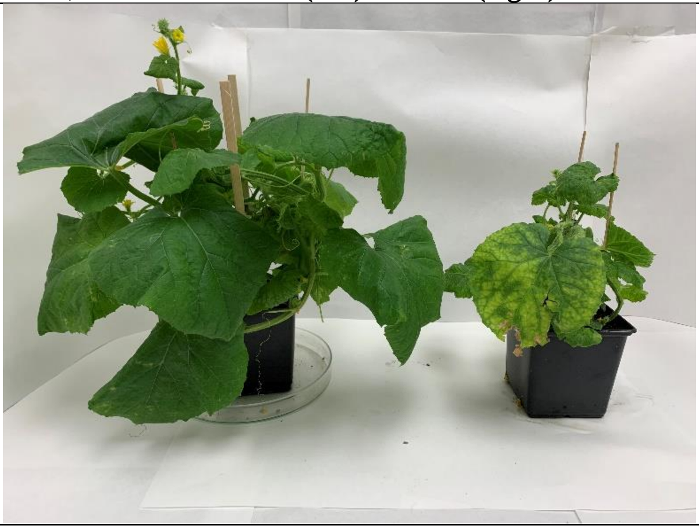

Healthy cv. Bazán Vs. healthy C311

Aphidius colemani behavior Changes Depending on Volatile Organic Compounds Emitted by Plants Infected with Viruses with Different Modes of Transmission. Clemente-Orta, G., Cabello, A., Garzo, E., Moreno, A., Fereres, A.

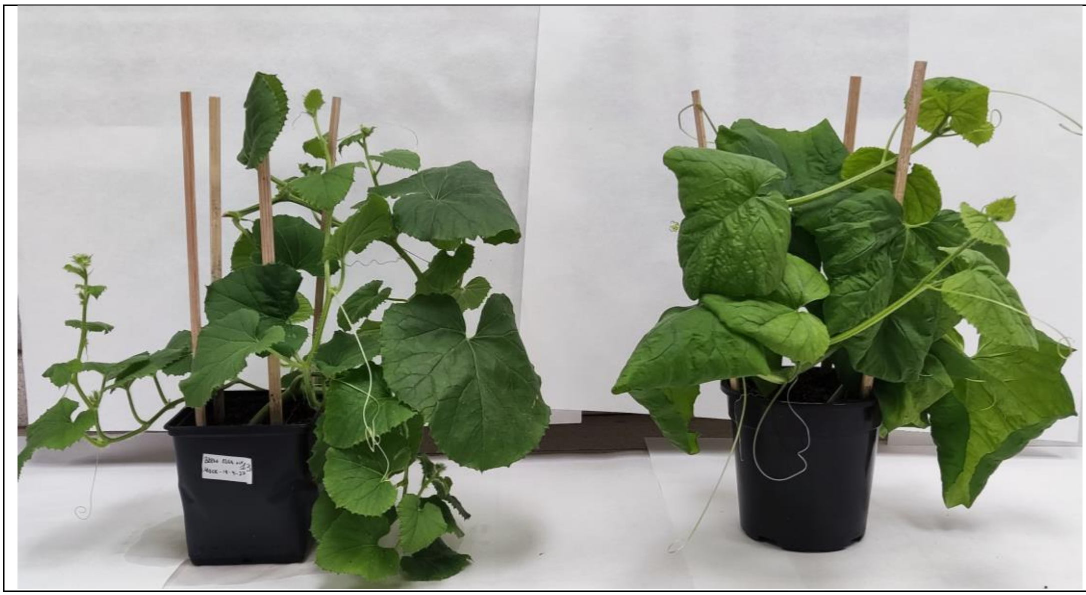

Supplement: Supplementary file 1 [file insects-15-00092-s001.zip › insects-2831249-supplementary.pdf]
